# Habitat‐mediated timing of migration in polar bears: an individual perspective

**DOI:** 10.1002/ece3.2233

**Published:** 2016-06-26

**Authors:** Seth G. Cherry, Andrew E. Derocher, Nicholas J. Lunn

**Affiliations:** ^1^ Department of Biological Sciences University of Alberta Edmonton AB T6G 2E9 Canada; ^2^ Environment and Climate Change Canada University of Alberta CW405 Biological Sciences Building Edmonton AB T6G 2E9 Canada

**Keywords:** GPS collars, migration, polar bears, sea ice, spatiotemporal scale, time‐to‐event models

## Abstract

Migration phenology is largely determined by how animals respond to seasonal changes in environmental conditions. Our perception of the relationship between migratory behavior and environmental cues can vary depending on the spatial scale at which these interactions are measured. Understanding the behavioral mechanisms behind population‐scale movements requires knowledge of how individuals respond to local cues. We show how time‐to‐event models can be used to predict what factors are associated with the timing of an individual's migratory behavior using data from GPS collared polar bears (*Ursus maritimus*) that move seasonally between sea ice and terrestrial habitats. We found the concentration of sea ice that bears experience at a local level, along with the duration of exposure to these conditions, was most associated with individual migration timing. Our results corroborate studies that assume thresholds of >50% sea ice concentration are necessary for suitable polar bear habitat; however, continued periods (e.g., days to weeks) of exposure to suboptimal ice concentrations during seasonal melting were required before the proportion of bears migrating to land increased substantially. Time‐to‐event models are advantageous for examining individual movement patterns because they account for the idea that animals make decisions based on an accumulation of knowledge from the landscapes they move through and not simply the environment they are exposed to at the time of a decision. Understanding the migration behavior of polar bears moving between terrestrial and marine habitat, at multiple spatiotemporal scales, will be a major aspect of quantifying observed and potential demographic responses to climate‐induced environmental changes.

## Introduction

Variation in animal distribution is largely a consequence of how species respond to temporal distributions of key resources. Measurements of population distributions relative to static or dynamic habitat characteristics can be achieved at numerous scales. Quantifying how and when animal populations respond to seasonal environmental shifts or long‐term habitat change often involves measurements of interacting factors over large geographic areas or landscapes (Stenseth et al. 2002; Parmesan et al. 2005; Hone and Clutton‐Brock 2007). Once acquired, baseline knowledge of landscape‐scale ecological relationships can be useful for assessing implications of global or regional environmental change in relation to population viability and distribution (Thomas et al. 2004; Austin and Rehfisch 2005; Van De Pol et al. 2010). However, finer‐scale mechanistic relationships between individuals and their local environment may differ from those measured at landscape scales, especially in heterogeneous habitats (Wiens 1989; Kuefler and Haddad 2006; Murray et al. 2008; Tøttrup et al. 2012). Studies focusing on individuals within a population and their local environment can therefore assist in determining how behavioral responses to immediate surroundings actually shape the overall distribution and evolution of a species. For example, understanding the behavioral mechanisms behind population‐level responses to environmental change, such as those observed during habitat‐mediated migration events, requires knowledge of how individuals react to local environmental cues.

In its simplest form, migration is a round‐trip movement between isolated areas at different times of the year (Ball et al. 2001; Berger 2004). Migratory phenomena are commonly observed in environments characterized by highly variable conditions. Both short‐ and long‐distance migrations are common in the Arctic, where seasonal shifts in distribution occur in a wide range of animals including zooplankton (Fischer and Visbeck 1993), fish (Grainger 1953), birds (Johnson and Herter 1990), terrestrial mammals (Fancy et al. 1989), and marine mammals (Laidre et al. 2008). For many of these species, migration phenology is variable among groups within a population (i.e., flocks, herds, or pods). In contrast, species such as the polar bear (*Ursus maritimus*) typically do not display gregarious behavior and therefore may have higher degrees of individual variability in the timing of migration events.

**Figure 1:**
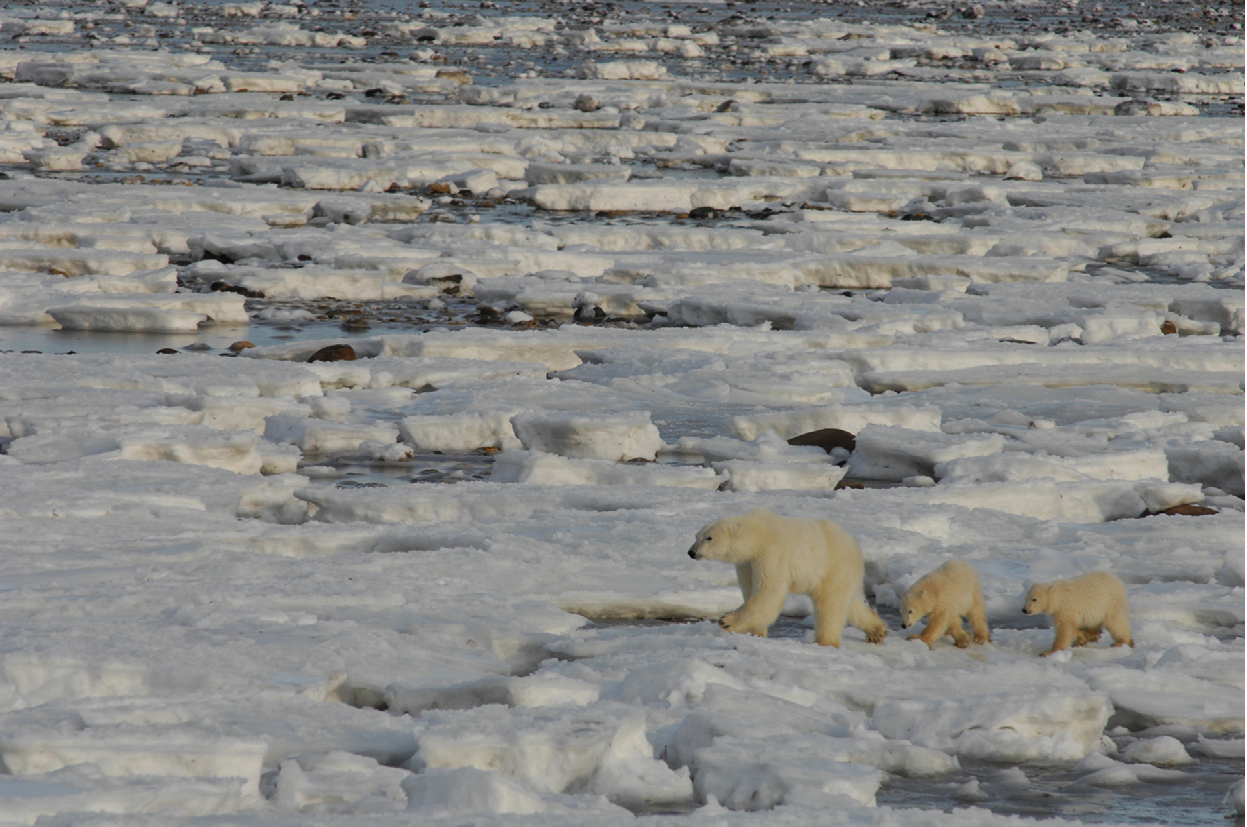
Collared female polar bear and her cubs walking on sea ice in western Hudson Bay, Canada.

The intra‐annual distribution of polar bears changes throughout their range in response to seasonal environmental conditions (Stirling et al. 1999; Mauritzen et al. 2003; Durner et al. 2009; Cherry et al. 2013). In some parts of their range, polar bears exhibit annual migrations involving movement from marine to terrestrial habitat that coincide with sea ice breakup during the summer thaw (Fig. 1 – Stirling et al. 1999, 2004; Schliebe et al. 2008; Rode et al. 2012; Cherry et al. 2013). In some of these regions, polar bear movement from sea to land is an obligatory migration caused by a complete melting of the ice platform they use for hunting, mating, and traveling (e.g., Derocher and Stirling 1990). In other areas, migration to land is facultative because summer use of offshore multiyear sea ice is a potential alternate when seasonal ice melts (Schliebe et al. 2008). The timing of sea ice melt, and thus migration to land, has been associated with polar bear body condition, survival, and reproduction (Derocher and Stirling 1995; Stirling et al. 1999; Regehr et al. 2007; Molnár et al. 2010, 2014). Although the interannual variation in timing of polar bear arrival ashore has been predicted by landscape‐scale indices of ice breakup (Stirling et al. 1999; Stirling and Parkinson 2006; Cherry et al. 2013), little is known about individual responses to localized and finer‐scale sea ice dynamics during the summer melt. Given the heterogeneity of sea ice habitat, particularly during breakup (Gagnon and Gough 2005), it is unlikely that ice conditions measured at landscape or regional scales represent what individual bears are exposed to at local scales. In addition, observed intrapopulation variation in the timing of annual arrival ashore (Stirling et al. 1999; Cherry et al. 2013; Sahanatien et al. 2015) is suggestive of an underlying mechanistic relationship between sea ice melt and individual polar bear migration at finer spatial scales than have been measured. Quantifying the behavioral responses of polar bears to disappearing ice will prove useful when predicting ice concentration thresholds for suitable habitat under climate change scenarios (e.g., Durner et al. 2009; Hunter et al. 2010; Regehr et al. 2010) and also provide an understanding of mechanisms affecting the distribution of the species.

One means of exploring causes of variation in the timing of ecological events is through individual‐based time‐to‐event modeling, which is more commonly known for its applications to survival analysis. Time‐to‐event models have been applied in ecological research to quantify sources of variation in kill rates for predator–prey systems (Merrill et al. 2010; McPhee et al. 2012) and the timing of various migratory events or behavioral responses (Bauer et al. 2008; Fieberg and DelGiudice 2008; Fieberg et al. 2008; Merkle et al. 2013). These techniques analyze the probability of an event occurring in relation to duration of exposure to various levels of potential explanatory factors rather than simply the conditions present when the event occurs (Hosmer and Lemeshow 1999; Therneau and Grambsch 2000; Cleves et al. 2002). Therefore, time‐to‐event models are useful for examining the mechanisms behind individual polar bear migratory behavior because a bear's decision to migrate is likely dependent upon both the ice conditions they experience before heading for land and the duration of exposure to these conditions.

In this study, we used time‐to‐event models to explore how exposure of individual polar bears to varying environmental conditions influences the timing of decisions to migrate from marine to terrestrial habitats in Hudson Bay, Canada. Based on findings of population‐level migration events (Stirling et al. 1999; Cherry et al. 2013), we hypothesized that both concentration and rate of change of sea ice experienced by individual bears at local scales would be factors in their decisions to migrate for shore. However, we were specifically interested in examining whether observed correlations between seascape environmental factors and population‐level movements differed from those involving local environmental factors and individual migration behavior. We also tested whether individual factors such as the distance a polar bear was from land at a given time or age and reproductive status explained variation in the timing of migration.

## Methods

Polar bears in the western Hudson Bay subpopulation show strong site fidelity to summering areas in northeastern Manitoba when the sea ice melts (Derocher and Stirling 1990; Stirling et al. 2004; Cherry et al. 2013). The bears use a large part of Hudson Bay when it is ice covered with home ranges averaging over 350,000 km^2^ during our study (McCall et al. 2015). Hudson Bay is a large inland sea comprising an area over 840,000 km^2^ that undergoes an annual cryogenic cycle with sea ice freeze‐up starting in late October, ice cover reaching maximum extent in December, followed by a late‐spring/summer melt and the Bay becoming ice free by August (Markham 1986; Gagnon and Gough 2005). Driven by large‐scale atmospheric patterns, sea ice formation and melt dynamics vary throughout the Bay and between years (Wang et al. 1994a; Mysak et al. 1996).

**Figure 2:**
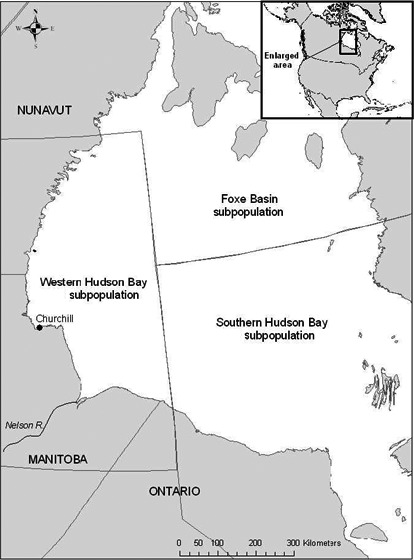
Map of the study area showing the boundaries for the western Hudson Bay and adjacent subpopulations. Polar bear captures occurred on land between the Nelson River and municipality of Churchill, Manitoba, Canada.

Polar bear captures occurred on land in autumn 2004–2008 between Churchill and the Nelson River (Fig. 2). Bears were located by helicopter and remotely immobilized via injection of tiletamine hydrochloride and zolazepam hydrochloride (Zoletil^®^, Laboratoires Virbac, Carros, France; Stirling et al. 1989). Animal care procedures were reviewed and approved by the University of Alberta BioSciences Animal Policy and Welfare Committee and the Environment Canada Prairie and Northern Region Animal Care Committee (2004PNR013, 2005PNR013, 2006PNR013, EC‐PN‐07‐013, EC‐PN‐08‐013). Global positioning system (GPS) Argos^®^ satellite‐linked collars (Telonics, Mesa, AZ) were deployed on adult females with either cubs‐of‐the‐year or 1‐year‐old cubs. Collars were programmed to obtain 1 GPS location every 4 h.

We used a time‐to‐event model to assess the relationship between the dates polar bears began a directional migration toward shore and both environmental factors and individual characteristics. Our time of origin (*t *=* *0) and onset of risk was defined as May 1 of each year because this is immediately before sea ice generally begins to melt in Hudson Bay (Saucier et al. 2004). Polar bears were considered to have entered a directional migration toward shore when the distance between their position and the location where they eventually arrived on land commenced a constant decline. Our model covariates describing environmental conditions experienced by individual bears included local sea ice concentration, local sea ice concentration rate of change, and distance to shore. Potential covariates describing individual characteristics included age and reproductive status (i.e., presence or absence of offspring during breakup).

Environmental covariate data for each GPS collar location were obtained using a geographic information system (ArcInfo 9.3, Environmental Systems Research Institute, Redlands, CA). Our time‐to‐event model used a daily time scale, and we subsampled the first available GPS location per bear each day. Local sea ice concentration at each bear location was approximated from daily Special Sensor Microwave/Imager (SSM/I) passive microwave data from the National Snow and Ice Data Center (Boulder, CO, http://nsidc.org/). Using a single GPS location per day ensured the temporal and spatial resolution of the collar data matched the SSM/I sea ice data. Mean springtime movement rate for adult female polar bears in western Hudson Bay is 31.2 km/day (Parks et al. 2006), and SSM/I data provide ice concentration at a resolution of 25 × 25 km cells. Daily sea ice rate of change was determined as the difference in local ice concentration values between a bear's current and previous location and was thus influenced by daily melting and moving of ice and habitat selection by the bear. Distance to shore, calculated as the straight‐line distance between a location and the nearest point on the Hudson Bay mainland, was included in the analysis because the probability of a bear beginning a directional migration toward shore may depend on their distance from land.

A vestigial premolar was extracted from each collared bear to determine age using cementum annuli (Calvert and Ramsay 1998). We hypothesized that experience and thus age may influence movements to shore during breakup. We further hypothesized presence or absence of offspring may affect timing of migration because offspring may be less efficient at traveling through highly fragmented ice and open water. Polar bears in western Hudson Bay typically have a 3‐year interbirth interval (Derocher and Stirling 1995), and unless a bear was re‐sighted, we used this interval to infer reproductive status. Cubs are born in November–December and offspring typically accompany their mothers until March–May after their second year (Lunn et al. 2004), and therefore, we assumed females and 2‐year‐old offspring had separated by the time they began directional movement toward shore. Reproductive status, inferred from status at the time of capture, was defined as alone or with yearlings because at the time of capture the previous August all collared bears either had cubs‐of‐the‐year or 1‐year‐old cubs.

To choose the best type of time‐to‐event model for our data (i.e., nonparametric, semi‐parametric, or any of numerous available parametric models), it was necessary to consider the potential shape of our underlying baseline hazard function (Cleves et al. 2002; Merrill et al. 2010). The shape of an underlying baseline hazard function describes the hazard rate of an event occurring throughout time in the absence of measured covariate effects (Kumar and Klefsjo 1994; Cleves et al. 2002). The hazard rate for polar bears migrating toward shore in the absence of large‐scale seasonal sea ice melting is likely constant with time because bears in northerly regions, with less drastic seasonal changes to environmental conditions, rarely travel to land (Thiemann et al. 2008). Pregnant females in these northerly regions often come to land to build maternity dens; however, maternity denning does not occur until autumn (Messier et al. 1992) and after our study period, which ended with arrival on shore. Additional evidence of a constant baseline hazard comes from observations in 1992 when sea ice in Hudson Bay melted much later than normal, due to the eruption of Mount Pinatubo in the Philippines, and polar bears responded by substantially delaying their migration to land (Stirling et al. 1999). This delayed migration in 1992 supports the idea that hazard rates for polar bears migrating to land are related to specific environmental conditions and would otherwise not change with time. Therefore, we chose to use a parametric underlying baseline hazard in the exponential form, which assumes hazard remains constant with time in the absence of covariate effects (Hosmer and Lemeshow 1999; Therneau and Grambsch 2000; Cleves et al. 2002).

Statistical analyses were performed in STATA 10 (Stata Corporation, College Station, TX). We used Akaike's information criterion analysis, corrected for small sample size (AICc), to evaluate relative support for exponential models examining effects of environmental covariates. All combinations of main effects involving environmental covariates were tested. We also examined 2‐way interactions for environmental covariates; however, interactions were only added to models including both corresponding main effects. Once the best‐fitting model using environmental covariates was identified, we used this model in a second stage of AICc analysis that examined combinations of proposed individual characteristics to determine whether model fit was improved.

We tested for collinearity among environmental covariates using a Pearson's correlation and no variable had |*r*| >0.65. We also visually inspected plots of Martingale residuals for each covariate to assess covariate functional forms and ensure our data met proportional hazard assumptions (Cleves et al. 2002). To test our assumption regarding an exponential baseline hazard, we assessed whether the overall hazard for our events varied solely with covariates rather than time (Cleves et al. 2002). We included time, measured as ordinal date, as a covariate in the global models from each AICc analysis to test whether time significantly explained observed variation in the overall hazard. In addition, we compared 95% confidence intervals of the coefficients in the global parametric models with equivalent semi‐parametric Cox proportional hazards models with the assumption that a well‐fitting parametric model should approximate the coefficients of a Cox proportional hazards model (Cleves et al. 2002; Merrill et al. 2010). We expressed the best‐fitting exponential model using proportional hazard metrics. Proportional hazard parameters describe the change in relative likelihood of polar bears beginning a directional migration toward shore, on any given day, per unit change in given environmental characteristics (Hosmer and Lemeshow 1999; Therneau and Grambsch 2000; Cleves et al. 2002).

We then compared the daily values for covariates in the best‐fitting model to corresponding values for environmental covariates determined at the regional level. This aspect of the analysis was carried out to quantify how environmental variation at the individual and local level may differ from regional means. Daily regional environmental covariates were determined using data and methods described in Cherry et al. (2013). Differences in local and regional daily environmental covariates were determined for each bear and expressed as absolute mean differences for all collared bears during various stages of spring sea ice breakup, which were defined as 10% ice concentration intervals beginning on May 1 of each year when Hudson Bay is mostly ice covered and ending when bears began directional movement toward land.

## Results

We deployed 59 GPS collars on 56 different polar bears but due to collar failures, our analyses used 21 migrations from the sea ice to land for 20 individuals. The mean age of the bears in our analysis was 17 years (range = 8–26 years). Based on our projection of reproductive status, 13 bears had 1‐year‐old cubs and 8 bears were alone during migration events. The mean date bears began to head for shore was July 13 (SE = 2.2 days, range = June 28–August 7). There was an average of 5.8 (SE = 0.9) days between the date polar bears began a directional migration toward shore and their first recorded location on land. When polar bears began a directional migration toward shore, they were a mean distance of 80.4 km (SE = 10.6, range = 4.4–178.5 km) from the coastline.

We found similar support (∆AICc <2) for the time‐to‐event model examining environmental covariates that used local sea ice concentration by itself and models that combined local sea ice concentration with either distance to shore or sea ice rate of change (Table 1). However, the model using only local sea ice concentration was chosen as the best‐fitting model because it had 1 less parameter than the 2 competing models with ∆AICc <2. Addition of various combinations of individual characteristics to the model using the sea ice covariate alone did not improve model fit (Table 2). Proportional hazard parameterization of the best model indicated the likelihood of bears heading for shore at a given time increased by a factor of 1.07 per percentage decrease in the daily local sea ice concentration (HR = 0.93, SE = 0.02, *P *<* *0.001, 95% CI = 0.90–0.96).

**Table 1:** Competing hypotheses from an exponential proportional hazards model evaluating what best explains variation in dates polar bears began their directional migration to shore between 2004 and 2008 in western Hudson Bay. Model comparisons are based on the Akaike's information criterion, corrected for small sample size (AICc). ∆AICc is the difference in AICc scores between different candidate models and the best model, and *w* is the Akaike weight or the weight of evidence that a model is the best approximating model given the data and the set of models considered. Covariates: sea ice concentration = iceconc; distance to shore = distshore; sea ice concentration rate of change = rate

Model	AICc	∆AICc	*w*
Iceconc	−20.67	0	0.39
Iceconc, distshore	−19.19	1.48	0.19
Iceconc, rate	−18.95	1.72	0.17
Iceconc, distshore, rate	−17.43	3.24	0.08
Iceconc, rate, iceconc*rate	−16.73	3.94	0.06
Iceconc, distshore, iceconc*distshore	−16.43	4.24	0.05
Iceconc, distshore, rate, iceconc*rate	−15.04	5.63	0.02
Iceconc, distshore, rate, distshore*rate	−14.46	6.21	0.02
Iceconc, distshore, rate, iceconc*distshore	−14.36	6.31	0.02
Iceconc, distshore, rate, iceconc*rate, distshore*rate	−11.58	9.09	0
Iceconc, distshore, rate, iceconc*distshore, iceconc*rate	−11.56	9.11	0
Iceconc, distshore, rate, iceconc*distshore, distshore*rate	−10.98	9.69	0
Iceconc, distshore, rate, iceconc*distshore, iceconc*rate, distshore*rate	−7.6	13.07	0
Distshore	39.49	60.16	0
Distshore, rate	41.85	62.52	0
Distshore, rate, distshore*rate	44.55	65.22	0
Rate	46.35	67.02	0
Null	44.34	65.01	0

**Table 2:** Competing hypotheses from an exponential proportional hazards model evaluating the best‐fitting environmental covariate model with combinations of individual parameters. Model comparisons are based on the Akaike's information criterion, corrected for small sample size (AICc). ∆AICc is the difference in AICc scores between different candidate models and the best model, and *w* is the Akaike weight or the weight of evidence that a model is the best approximating model given the data and the set of models considered. Covariates: sea ice concentration = iceconc; age = age; reproductive status = reprod

Model	AICc	∆AICc	*w*
Iceconc	−20.67	0	0.59
Iceconc, reprod	−18.35	2.32	0.18
Iceconc, age	−18.21	2.46	0.17
Iceconc, reprod, age	−15.61	5.06	0.05
Iceconc, reprod, age, reprod*age	−12.52	8.15	0.01
Null	44.34	65.01	0

Examination of the Martingale residuals indicated the functional forms of the covariates did not require transformation to meet proportional hazard assumptions for our exponential model (Cleves et al. 2002). Time, measured as ordinal date, was not significant when it was included in the environmental covariate global model (HR_time_ = 1.04, SE = 0.03, *P = *0.09, 95% CI = 0.99–1.10) or the global model assessing effects of individual characteristics (HR_time_ = 1.05 SE = 0.023, *P = *0.07), which supports our assumption of an underlying exponential baseline hazard. In addition, the coefficients from global exponential models had overlapping 95% confidence intervals with those of equivalent semi‐parametric Cox proportional hazards models, indicating that we attained well‐fitting parametric models.

**Figure 3:**
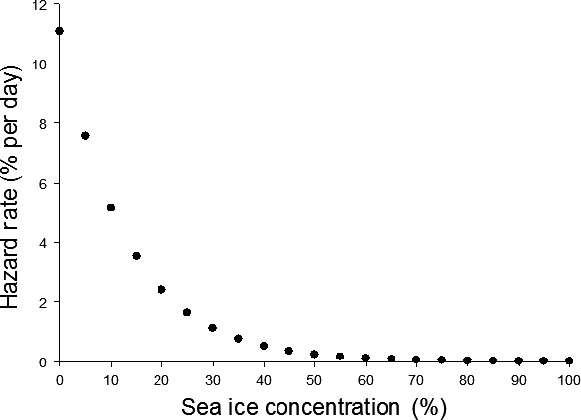
Predicted instantaneous hazard rate for daily ice concentrations (within 25 × 25 km cells) given in 5% intervals. Instantaneous hazard rates are expressed as the percentage of individuals migrating to shore per day and are conditional upon subjects having not already migrated. Predictions based on the best‐fitting exponential time‐to‐event model accounting for the variation in dates polar bears began their directional migration to shore between 2004 and 2008 in western Hudson Bay.

**Figure 4:**
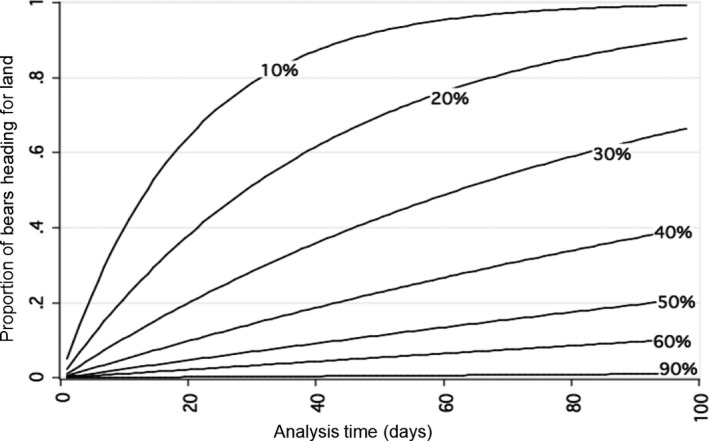
Estimated cumulative “failure” curves from the fitted exponential time‐to‐event model indicating the expected proportion of polar bears over time beginning a directional migration toward shore when continuously exposed to various daily sea ice concentrations (%).

To further examine how sea ice affects timing of polar bear migration toward shore, we estimated the instantaneous hazard function or daily rate of failure for various sea ice increments. It was possible to graph the daily rate of failure for various increments of covariate values independent of time because hazard functions in exponential time‐to‐event models “lack memory” and thus remain constant throughout time at a given covariate value (Cleves et al. 2002). The predicted instantaneous hazard rate or daily rate of “failure” appeared negligible when polar bears were exposed to daily local ice concentrations >60%; however, it increased rapidly as daily local ice concentration decreased below 60% (Fig. 3). We also estimated cumulative failure (1 minus “survival”) curves from our fitted model, which indicated the expected proportion of bears over time exhibiting directional migration toward shore when consistently exposed to given ice concentrations. The predicted proportion of polar bears heading for shore when continuously exposed to various local sea ice concentrations over time was highest for low daily ice concentrations and negligible for high daily ice concentrations (>60%) (Fig. 4).

**Figure 5:**
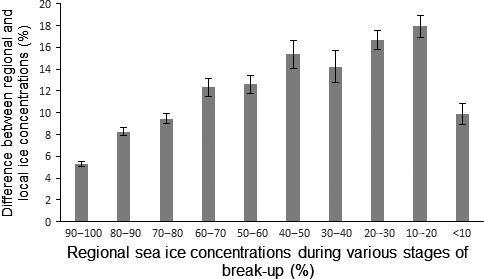
The absolute difference between daily regional sea ice concentrations and daily ice concentrations that individual polar bears in western Hudson Bay experience at the local scale. Differences are expressed as means (± SE) for collared bears during various stages of spring breakup. Stages of sea ice breakup are based on 10% intervals in the mean regional ice concentration starting on May 1.

The absolute differences between daily sea ice concentrations at the local and regional scales changed throughout various stages of spring breakup (Fig. 5). The absolute differences were lower (<10%) during early stages of breakup when regional ice concentrations were high (>70% regional ice concentration). Absolute differences between local and regional values increased and approached 20% during the “next to final” stages of breakup, which occurred when regional ice concentrations were 10–30%. The absolute differences between local and regional ice concentration values declined again when regional ice concentrations became < 10% during the final stages of breakup.

## Discussion

We found that sea ice concentration was the main factor affecting the timing of migration of individual bears from sea ice to land. Our results corroborate, to some degree, assumptions that a landscape‐scale threshold of >50% sea ice concentration is necessary for polar bear habitat (i.e., Schliebe et al. 2008; Durner et al. 2009; Gleason and Rode 2009; Hunter et al. 2010; Regehr et al. 2010). However, continued periods (e.g., days to weeks) of exposure to suboptimal ice concentrations at the local scale were required before the expected proportion of bears migrating toward shore increased substantially. For example, 40% of bears would be expected to have left for shore after just 10 days of being exposed to local sea ice concentrations of 10%, whereas <10% of bears would be expected to migrate to shore after being exposed to local sea ice concentrations of 50% for 10 days. In reality, the sea ice conditions that bears are exposed to from day to day will often differ and thus the likelihood of a bear migrating to land will increase by varying amounts each day as the sea ice melts and they accumulate experience and knowledge with regard to the depleting ice habitat. These results provide key insights into the mechanistic relationship between deteriorating sea ice at the local level and the decision individual polar bears make to abandon marine habitat and migrate to land. Recently, individual variation in habitat selection in female polar bears was found in the western Hudson Bay subpopulation (McCall et al. 2016) and supports our findings. Depending on the region, polar bears typically select for habitat with ice concentrations between 60% and 95% (Ferguson et al. 2001; Mauritzen et al. 2003; Durner et al. 2004, 2009; Pilfold et al. 2013), but have shown selection for ice concentrations as low as 10–60% in some areas and at certain times of the year (Arthur et al. 1996; Mauritzen et al. 2003; Durner et al. 2006). Even when our measures of ice concentration in 25 × 25 km cells were approaching zero, there may still have been small amounts of remnant ice that allowed for a refuge for some bears to delay their migration to land. During breakup in Hudson Bay, it may be energetically beneficial for polar bears to continue hunting on the sea ice as it continues to melt below optimal ice concentrations because they have a relatively short swim to land (Durner et al. 2009).

Previous research shows that landscape‐scale predictors of sea ice concentrations during breakup correlate with population‐level migration; however, the annual mean dates bears arrive ashore tend to be several weeks after these landscape metrics for breakup occur (e.g., Stirling et al. 1999; Cherry et al. 2013). These large‐scale predictors may differ from ice conditions that individual bears are experiencing in their immediate surroundings when they decide to abandon the marine environment, which likely explains the substantial variation in migration dates in our study (June 28–August 7). We found the degree of variation between daily regional and local sea ice concentrations differed somewhat among various stages of annual spring breakup. The highest variation between regional and local values occurred when regional ice concentrations were 10–30%, which likely corresponds to a time when the seascape is in a highly heterogeneous state. This stage of breakup also corresponds to a time when polar bears in western Hudson Bay are positioning themselves close to shore in anticipation of making a decision to migrate to land. Therefore, examining local level ice conditions for individual bears at this stage is a key component of understanding the mechanism that drives migration behavior.

We found the length of time individual bears are exposed to deteriorating local ice conditions also plays an important role in their decision to migrate to shore. We were able to quantify the explicit relationship between decreasing local ice concentrations during breakup and the expected proportion of bears migrating to land over time. Because animals are capable of accumulating and using knowledge about the landscapes they move through (Smouse et al. 2010; Merkle et al. 2013), understanding the causes of individual movement patterns requires quantifying relationships between the length of exposure to given environmental conditions and resulting behavioral responses. Here, we provide a time‐to‐event modeling approach that accounts for accumulated experiences over time (i.e., an individual's exposure to various local ice concentrations throughout the days leading up to the decision to migrate).

In contrast to research examining polar bear migration at the landscape scale (i.e., Cherry et al. 2013), we found that sea ice rate of change measured at the individual and local scale did not increase the predictability of the timing of individual polar bear migration. At a local scale, it appears as though polar bears only respond to the quantity of ice habitat available and length of exposure to suboptimal conditions rather than the day‐to‐day rate at which ice conditions fluctuate. These observations demonstrate how spatial and time scales at which environmental factors are measured can lead to significant differences in their perceived effects (Wiens 1989; Ciarniello et al. 2007; Pinto and Keitt 2008). Similarly, Johnson et al. (2002) show that measurements of various habitat variables influence caribou (*Rangifer tarandus*) movements at different spatial scales and suggest that understanding which scales animals respond to provides insight into mechanistic reasons for movement between habitat types. For polar bears, fine‐scale and daily measurements of sea ice rate of change may vary rapidly and temporarily based on localized wind events and habitat heterogeneity, making the rate at which ice changes at the local level less likely to influence the timing of individual migration. In contrast, the rate of sea ice change at the landscape scale is likely a good indicator of how soon the population's marine habitat will be completely ice free.

Our measurements of daily distance to shore also did not influence the predictability of a polar bear's migration toward land. Sea ice in Hudson Bay mostly occurs along the western and southwestern coasts during later stages of breakup (Wang et al. 1994b; Gough and Allakhverdova 1999; Saucier et al. 2004), and western Hudson Bay polar bears show high degrees of seasonal fidelity to specific coastal regions in Manitoba during the summer ice‐free period (Derocher and Stirling 1990; Lunn et al. 2004; Cherry et al. 2013). Thus, polar bears likely have a propensity during breakup to use sea ice habitat in proximity to these coastal regions to avoid coming ashore in unfamiliar areas (Stirling et al. 2004; Cherry et al. 2013). In our study, the measurement for distance to shore probably did not affect variation in the timing of migration because most bears were close to land and within the distances that polar bears are capable of swimming (Pagano et al. 2012; Pilfold et al. 2016) when they began a directional move toward shore. Long‐distance swimming (>50 km) in Hudson Bay is uncommon relative to other subpopulations and is likely related to the distribution of sea ice at breakup (Pilfold et al. 2016). However, body condition, which was not assessed in our study, could affect the timing of migration for individuals because the amount of fat reserves they have could influence the energetic cost‐benefit trade‐off of staying longer on the ice versus abandoning their access to seals and heading for land.

Neither age nor reproductive status affected variation in timing of directional movement toward land. Our findings are similar to other studies that suggest female polar bears with older offspring do not have significantly decreased mobility, even during the breakup period (Ferguson et al. 2001; Parks et al. 2006; but see Amstrup et al. 2000). Nevertheless, physiological constraints of dependent offspring in other marine mammals and ursids can impede movement in certain habitats (White et al. 2000; Loseto et al. 2006) and an assessment of family groups that include cubs‐of‐the‐year may be particularly important to understand how dependent offspring affect migration. Additionally, more detailed observations of cub presence or absence using proximity sensor transmitters would increase the certainty of the presence or absence of offspring with females at various times of the year.

In our study, we demonstrate the importance of examining how individuals respond to localized daily environmental conditions and thus provide a more mechanistic understanding of spatial dynamics at the population level. For western Hudson Bay polar bears, continued monitoring of the relationship between exposure length to various environmental conditions and individual migration timing may be a key aspect of quantifying behavioral changes as a result of future climate change. Additional monitoring of polar bear movement behavior with improved GPS collar technology will also allow for an increased sample size and provide an opportunity to incorporate a higher number of potential environmental factors into time‐to‐event models. The longer‐term projections for sea ice conditions in Hudson Bay suggest the duration of the on‐land period will increase with negative demographic consequences (Molnár et al. 2010, 2011, 2014; Castro de la Guardia et al. 2013). Although there is evidence of terrestrial foraging by polar bears (Russell 1975; Derocher et al. 1993; Gormezano and Rockwell 2013), they mostly fast and rely on stored fat reserves for energy while on land and terrestrial resources are inadequate to offset lost hunting on the sea ice (Ramsay and Stirling 1988; Hobson et al. 2009; Rode et al. 2015). Therefore, understanding and monitoring migration patterns between marine and terrestrial habitats will be a major component of quantifying population demographic responses and trends associated with a changing climate.
